# Short‐Term Morbidity and Mortality after Distal Femur Open Reduction Internal Fixation in the Geriatric Population

**DOI:** 10.1111/os.14124

**Published:** 2024-06-04

**Authors:** Jasmin Vargas, Mark A. Plantz, Erik B. Gerlach, Tyler Compton, Jennings Dooley, Clayton Welsh, Bennet Butler

**Affiliations:** ^1^ Department of Orthopaedic Surgery Northwestern University Chicago Illinois USA

**Keywords:** Distal femur fracture, Geriatric, Orthopaedic surgery, NSQIP

## Abstract

**Objective:**

Distal femur fractures remain a significant cause of morbidity and mortality for elderly patients. There is a lack of large population studies investigating short‐term outcomes after distal femur c in elderly patients. The purpose of this study is to assess the incidence of and risk factors for various short‐term outcomes after distal femur open reduction internal fixation (ORIF) in the geriatric population.

**Methods:**

The American College of Surgeons’ National Surgical Quality Improvement Program (NSQIP) database was used to identify all primary distal femur ORIF cases in patients 60+ years old between January 1, 2015 and December 31, 2020 using Current Procedural Terminology (CPT) codes 27511, 27513, and 27514. Demographic, medical, and surgical variables were extracted for all patients. Propensity score matching was used to match cases in the two age groups based on various demographic and medical comorbidity variables. Several 30‐day outcome measures were compared between the 60–79‐year‐old and 80+‐year‐old groups both before and after matching. Subsequent multivariate logistic regression was used to identify independent risk factors for 30‐day outcome measures in the matched cohort.

**Results:**

A total of 2913 patients were included in the final cohort: 1711 patients in the 60–79‐year‐old group and 1202 patients in the 80+‐year‐old group. Most patients were female (*n* = 2385; 81.9%). Prior to matching, the older group had a higher incidence of 30‐day mortality (1.9% vs. 6.2%), readmission (3.7% vs. 9.7%, *p* = 0.024), and non‐home discharge (74.3% vs. 89.5%, *p* < 0.001). Additionally, the older group had a higher rate of blood loss requiring transfusion (30.9% vs. 42.3%, *p* < 0.001) and medical complications (10.4% vs. 16.4%, *p* < 0.001), including myocardial infarction (0.7% vs. 2.7%, *p* < 0.001), pneumonia (2.7% vs. 4.6%, *p* = 0.008), and urinary tract infection (4.1% vs. 6.1%, *p* = 0.0188). After matching, the older group consistently had a higher incidence of mortality, non‐home‐discharge, blood loss requiring transfusion, and myocardial infarction. Various independent risk factors were identified for 30‐day morbidity and mortality, including American Society of Anesthesiologists (ASA) classification, body mass index (BMI) status, operative duration, and certain medical comorbidities.

**Conclusion:**

Geriatric patients undergoing distal femur ORIF are at significant risk for 30‐day morbidity and mortality. After matching, octogenarians and older patients specifically are at increased risk for mortality, non‐home discharge, and surgical complications compared to patients aged 60–79 years old. Multiple factors, such as BMI status, ASA classification, operative time, and certain medical comorbidities, are independently associated with poor 30‐day outcomes.

## Graphical abstract

Geriatric patients undergoing distal femur open reduction internal fixation are at significant risk for 30‐day morbidity and mortality. After controlling for baseline medical comorbidity, 80+‐year‐olds are at increased risk for mortality and surgical complications compared to patients aged 60–79 years old.

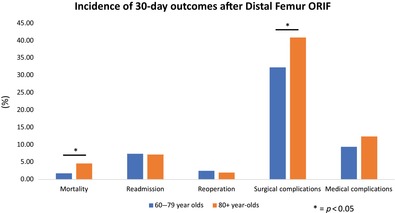

## Introduction

Distal femur fractures are relatively rare injuries, comprising roughly 0.4% of all fractures and 3% of femur fractures.^1^ The overall incidence has been estimated at 8.7/100,000 per year.^2^ Similar to many fractures, there is a classic bimodal distribution with a peak for men in their 30s and another peak for geriatric patients.^3^ After the age of 60 years, a significant increase in the incidence of these injuries has been observed, with a significant female predominance.^2^

Within the geriatric population, distal femur fractures treated with open reduction internal fixation (ORIF) are associated with significant risks; the 30‐day morbidity and mortality rates after distal femur ORIF are comparable to those seen after femoral neck fracture fixation.^4^ Distal femur fractures can involve osteoporotic bone, articular extension, and significant comminution, which can necessitate weight‐bearing restrictions and further complicate postoperative rehabilitation.^5^ The risks of non‐union, loss of mobility, and lack of independence after distal femur ORIF are significant.^6^ Furthermore, the economic burden of these injuries to patients and the health system are substantial.^7^

Despite the complexity and burden of these injuries, there is a lack of literature investigating morbidity after distal femur ORIF in the geriatric population. Most of the existing literature on the topic has been limited by small sample sizes. The purpose of this study is to: (i) investigate the incidence of various short‐term outcome measures (including morbidity, mortality, readmission, reoperation, and various medical and surgical complications) after distal femur ORIF in the geriatric population; (ii) utilize exact score matching to control for differences in patient demographic and medical comorbidities between groups; and (iii) identify independent variables associated with the various short‐term outcome measures using multivariate logistic regression.

## Methods

### Data Source

The American College of Surgeons’ National Surgical Quality Improvement Program (ACS NSQIP) database was the source of data for this study.^8^ The ACS NSQIP reports various 30‐day outcome measures after a variety of surgical procedures from participating institutions across the nation.^8^ Trained clinical reviewers from each participating institution collect and submit data from over 700 participating institutions. Patient demographics, medical comorbidities, and operative data are also provided in a deidentified manner.

The American College of Surgeons’ NSQIP database was used to identify all primary distal femur ORIF cases in patients 60+ years old between January 1, 2015 and December 31, 2020. Cases were identified using current procedural terminology (CPT) codes 27511, 27513, and 27514. Any cases with concurrent procedures or incomplete data were excluded from the final cohort. Patients were categorized into two groups based on age: (i) 60–79 years old and (ii) 80+ years old.

### Variables of Interest

Patient demographics and other variables, including sex, age, body mass index (BMI), American Society of Anesthesiologists (ASA) class, and several medical comorbidities, were extracted for each case. BMI was categorized into the following groups: underweight (<18.5 kg/m^2^), normal (18.5–24.9 kg/m^2^), overweight (25.0–29.9 kg/m^2^), obesity class I (30.0–34.9 kg/m^2^), obesity class II (35.0–39.9 kg/m^2^), and obese class III (40.0+ kg/m^2^). Medical comorbidities included diabetes (insulin vs. non‐insulin dependent), chronic obstructive pulmonary disease (COPD), congestive heart failure (CHF), ascites, smoking, hypertension, renal failure, dialysis use, chronic steroid use, cancer, bleeding disorders, and blood transfusion required in 72 h prior surgery (e.g. preoperative blood transfusion). Surgical variables included operative time, reported in minutes. Operative time was also reported categorically into two groups: operative time less than 120 min and greater or equal to 120 min.

### Exact Score Matching

Exact score matching was used to match patients in the two age groups. Specifically, the two groups were paired in a 1:1 manner using a balanced, nearest neighbor approach with exact matching of the following categorical variables: sex, BMI group, ASA class, the presence of several medical comorbidities (diabetes, COPD, CHF, ascites, smoking, hypertension, renal failure, dialysis use, chronic steroid use, cancer, bleeding disorders, and blood transfusion required in 72 h prior surgery), and operative time.

Both prior to and after matching, the rate of various 30‐day outcome measures were compared between the two groups. The outcomes of interest included mortality, readmission, reoperation, and non‐home discharge. Surgical complications included superficial surgical site infection (SSI), deep SSI, wound infection, dehiscence, and blood loss requiring transfusion. Medical complications included pneumonia, unplanned intubation, pulmonary embolism, ventilator use over 48 hours postoperatively, renal insufficiency, renal failure, urinary tract infection, cerebrovascular accident, cardiac arrest, myocardial infarction, deep venous thromboembolism, systemic sepsis, and septic shock.

### Statistical Analysis

Categorical variables were compared using *χ* ^2^‐tests or Fisher's exact tests where appropriate. Continuous variables were compared using unpaired *t*‐tests. After matching, multivariate logistic regression was used to identify demographic and other patient variables that were associated with the various outcome measures of interest. Statistical significance was defined as *p* < 0.05. All statistical analyses were completed using IBM SPSS Version 24.0 (Armonk, NY: IBM Corp).

## Results

### Cohort Demographics and Comorbidities

Based on inclusion criteria, the study cohort comprised 1711 patients in the 60–79‐year‐old group and 1202 patients in the 80+‐year‐old group undergoing distal femur ORIF. In the 60–79‐year‐old group, 20.2% were men and 79.8% were women, while in the 80+‐year‐old group, 15.2% were men and 84.8% were women (*p* < 0.001). The distribution of BMI revealed significant differences between the two age groups, with the older cohort demonstrating a higher prevalence of underweight patients and lower prevalence of obesity classes I, II, and III (*p* < 0.001). Furthermore, the 80+‐year‐olds were more likely to have an ASA class of IV (*p* < 0.001).

As summarized in Table 1, notable variations in the rate of comorbidities were found between the age groups. In summary, the 80+‐year‐old cohort had higher rates of CHF, hypertension, bleeding disorders, and a preoperative blood transfusion (*p* < 0.05). The younger cohort had higher rates of both non‐insulin and insulin‐dependent diabetes, smoking, dialysis use, cancer, and chronic steroid use (*p* < 0.05). The younger cohort had slightly longer operative times on average (112.0 ± 53.8 min vs. 105.3 ± 47.5 min, *p* < 0.001). The younger cohort also had a higher percentage of patients with operative time greater than or equal to 120 min (35.2% vs. 30.8%, *p* = 0.015).

**TABLE 1:** Demographics, comorbidities, and surgical variables of the cohort.

Demographic variable	60–79 years old (*n* = 1711)		80+ years old (*n* = 1202)		*χ* ^2^/*T* value^a^	*p*
Sex						
Male	345	(20.2%)	183	(15.2%)	11.605	**<0.001**
Female	1366	(79.8%)	1019	(84.8%)		
BMI (kg/m^2^)						
Underweight	43	(2.5%)	60	(5.0%)	12.716	**<0.001**
Normal	312	(18.2%)	413	(34.4%)	98.193	**0.006**
Overweight	442	(25.8%)	366	(30.4%)	7.507	**0.125**
Obesity class I	345	(20.2%)	215	(17.9%)	2.357	**<0.001**
Obesity class II	244	(14.3%)	103	(8.6%)	21.796	**<0.001**
Obesity class III	325	(19.0%)	45	(3.7%)	148.096	**<0.001**
Comorbidities						
Diabetes						
No	1145	(66.9%)	975	(81.1%)	71.804	**<0.001**
Non‐insulin dependent	248	(14.5%)	123	(10.2%)	11.536	**<0.001**
Insulin dependent	318	(18.6%)	104	(8.7%)	56.235	**<0.001**
Smoking	275	(16.1%)	42	(3.5%)	115.179	**<0.001**
COPD	171	(10.0%)	103	(8.6%)	1.683	0.195
Ascites	3	(0.2%)	2	(0.2%)	0.003	0.954
Congestive heart failure	50	(2.9%)	68	(5.7%)	13.587	**<0.001**
Hypertension	1169	(68.3%)	920	(76.5%)	23.496	**<0.001**
Renal failure	19	(1.1%)	13	(1.1%)	0.005	0.941
Dialysis	69	(4.0%)	21	(1.7%)	12.318	**<0.001**
Cancer	33	(1.9%)	6	(0.5%)	10.923	**<0.001**
Chronic steroid use	108	(6.3%)	44	(3.7%)	10.036	**0.002**
Bleeding disorder	226	(13.2%)	224	(18.6%)	15.920	**<0.001**
Preoperative blood transfusion	126	(7.4%)	135	(11.2%)	12.944	**<0.001**
ASA class						
Class 1 (no disturbance)	16	(0.9%)	1	(0.1%)	8.832	**0.003**
Class 2 (mild disturbance)	360	(21.0%)	140	(11.6%)	43.811	**<0.001**
Class 3 (severe disturbance)	1094	(63.9%)	795	(66.1%)	1.499	0.221
Class 4 (life threatening)	241	(14.1%)	261	(21.7%)	28.805	**<0.001**
Class 5 (Moribund)	0	(0.0%)	3	(0.2%)	–	–
Operative time						
Total operative time	112.0	±53.8^b^	105.3	± 47.5^b^	3.498	**<0.001**
Under 120 min	1109	(64.8%)	831	(69.1%)	5.920	**0.015**
Over 120 min	602	(35.2%)	371	(30.8%)	5.920	**0.015**

*Note*: *p*‐values less than 0.001 were outputted as <0.001 on the SPSS statistical package (in bold).

^a^ *χ* ^2^ reported for *χ* ^2^‐tests (categorical variables) and *t* reported for *t*‐tests (continuous variables).

^b^ Reported as mean ± standard deviation.

### Pre‐Matching 30‐Day Outcome Measures

In the younger cohort, readmission occurred in 7.3%, reoperation in 2.8%, non‐home discharge in 74.3%, and death in 1.9%. For the 80+‐year‐old group, these rates were 9.7%, 2.3%, 89.5%, and 6.2%, respectively. The older cohort showed significantly higher rates of readmission (*p* = 0.024) and mortality (*p* < 0.001) prior to matching.

The older group exhibited higher rates of overall medical complications (31.7% vs. 43.2%, *p* < 0.001), including pneumonia (2.7% vs. 4.6%, *p* = 0.008), urinary tract infection (4.1% vs. 6.1%, *p* = 0.018), and myocardial infarction (0.7% vs. 2.7%, *p* < 0.001). No significant differences were observed in unplanned intubation, pulmonary embolism, ventilator use >48 h, renal insufficiency and failure, cerebrovascular accident, cardiac arrest, deep venous thromboembolism, systemic sepsis, septic shock, and *Clostridioides difficile* infection (*p* > 0.05).

Surgical complications were observed in 31.7% of patients in the 60–79‐year‐old group, with the most common being blood loss requiring transfusion (30.9%). In the 80+‐year‐old group, overall surgical complications occurred in 43.2% of patients, with blood loss requiring transfusion being the most prevalent complication (42.3%). The older cohort had a significantly higher rate of postoperative blood transfusion (30.9% vs. 42.3%, *p* < 0.001) but otherwise similar rates of surgical complications, including surgical site infections, dehiscence, and wound infections (*p* > 0.05) (Table 2).

**TABLE 2:** Thirty‐day outcome measures and complications.

Outcome variable	60–79 years old (*n* = 1711)		80+ years old (*n* = 1202)		*χ* ^2^	*p*
Readmission	125	(7.3%)	116	(9.7%)	5.116	0.024
Reoperation	48	(2.8%)	28	(2.3%)	0.629	0.428
Non‐home discharge	1272	(74.3%)	1076	(89.5%)	103.993	**<0.001**
Mortality	33	(1.9%)	74	(6.2%)	35.664	**<0.001**
Surgical complications						
Overall	542	(31.7%)	519	(43.2%)	40.326	**<0.001**
Superficial surgical site infection	15	(0.9%)	14	(1.2%)	0.594	0.441
Wound infection	5	(0.3%)	3	(0.2%)	0.047	0.829
Deep surgical site infection	3	(0.2%)	0	(0.0%)	2.110	0.273
Dehiscence	3	(0.2%)	5	(0.4%)	1.493	0.287
Blood loss requiring transfusion	528	(30.9%)	508	(42.3%)	40.065	**<0.001**
Medical complications						
Overall	178	(10.4%)	197	(16.4%)	22.556	**<0.001**
Pneumonia	47	(2.7%)	55	(4.6%)	6.988	**0.008**
Unplanned intubation	27	(1.6%)	22	(1.8%)	0.272	0.602
Pulmonary embolism	18	(1.1%)	23	(1.9%)	3.776	0.052
Ventilator >48 h	15	(0.9%)	12	(1.0%)	0.114	0.736
Renal insufficiency	7	(0.4%)	1	(0.1%)	2.738	0.151
Renal failure	8	(0.5%)	4	(0.3%)	0.313	0.771
Urinary tract infection	71	(4.1%)	73	(6.1%)	5.559	**0.018**
Cerebrovascular accident	7	(0.4%)	11	(0.9%)	2.944	0.086
Cardiac arrest	15	(0.9%)	11	(0.9%)	0.012	0.913
Myocardial infarction	12	(0.7%)	33	(2.7%)	19.395	**<0.001**
Deep venous thromboembolism	10	(0.6%)	14	(1.2%)	2.909	0.088
Systemic sepsis	16	(0.9%)	14	(1.2%)	0.365	0.546
Septic shock	9	(0.5%)	14	(1.2%)	3.677	0.055
C. diff infection	9	(0.5%)	7	(0.6%)	0.046	0.977

*Note*: *p*‐values less than 0.001 were outputted as <0.001 on the SPSS statistical package (in bold).

### Cohort Matching

As summarized in Table 3, the matched cohort included 607 patients in both the 60–79‐year‐old and 80+‐year‐old groups. The distribution of sex and BMI in this cohort demonstrated no significant differences between the two age groups (*p* > 0.05). Men comprised 14.3% and women constituted 85.7% in both groups. The prevalence of comorbidities, including diabetes, smoking, COPD, ascites, congestive heart failure, renal failure, dialysis, cancer, chronic steroid use, bleeding disorder, and preoperative blood transfusion were the same in both groups. Further, the distribution of ASA classification also exhibited no significant variations between the age groups. The operative times were similar between groups after matching.

**TABLE 3:** Demographics, comorbidities, and surgical variables of the cohort after matching.

Demographic variable	60–79 years old (*n* = 607)	80+ years old (*n* = 607)	*χ* ^2^/*T* value^a^	*p*
Sex				
Male	87 (14.3%)	87 (14.3%)	–	>0.999
Female	520 (85.7%)	520 (85.7%)		
BMI (kg/m^2^)				
Underweight	18 (3.0%)	18 (3.0%)	–	>0.999
Normal	148 (24.4%)	148 (24.4%)	–	>0.999
Overweight	197 (32.5%)	197 (32.5%)	–	>0.999
Obesity class I	146 (24.1%)	146 (24.1%)	–	>0.999
Obesity class II	69 (11.4%)	69 (11.4%)	–	>0.999
Obesity class III	29 (4.8%)	29 (4.8%)	–	>0.999
Comorbidities				
Diabetes				
No	477 (78.6%)	477 (78.6%)	–	>0.999
Non‐insulin dependent	64 (10.5%)	64 (10.5%)	–	>0.999
Insulin dependent	66 (10.9%)	66 (10.9%)	–	>0.999
Smoking	20 (3.3%)	20 (3.3%)	–	>0.999
COPD	16 (2.6%)	16 (2.6%)	–	>0.999
Ascites	1 (0.2%)	1 (0.2%)	–	>0.999
Congestive heart failure	5 (0.8%)	5 (0.8%)	–	>0.999
Hypertension	440 (72.5%)	440 (72.5%)	–	>0.999
Renal failure	1 (0.2%)	1 (0.2%)	–	>0.999
Dialysis	5 (0.8%)	5 (0.8%)	–	>0.999
Cancer	0 (0.0%)	0 (0.0%)	–	>0.999
Chronic steroid use	16 (2.6%)	16 (2.6%)	–	>0.999
Bleeding disorder	63 (10.4%)	63 (10.4%)	–	>0.999
Pre‐op blood transfusion	27 (4.4%)	27 (4.4%)	–	>0.999
ASA class				
Class 1 (No disturbance)	1 (0.2%)	1 (0.2%)	–	>0.999
Class 2 (Mild disturbance)	118 (19.4%)	118 (19.4%)	–	>0.999
Class 3 (Severe disturbance)	426 (70.2%)	426 (70.2%)	–	>0.999
Class 4 (Life threatening)	62 (10.2%)	62 (10.2%)	–	>0.999
Class 5 (Moribund)	0 (0.0%)	0 (0.0%)	–	>0.999
Operative time				
Total operative time	107 ± 47.3^b^	107 ± 47.0^a^	0.293	0.769
<120 min	415 (68.4%)	415 (68.4%)	–	>0.999
120+ min	192 (31.6%)	193 (31.6%)	–	>0.999

^a^ *χ* ^2^ reported for *χ* ^2^‐tests (categorical variables) and *t* reported for *t*‐tests (continuous variables).

^b^ Reported as mean ± standard deviation.

### Post‐Matching 30‐Day Outcomes and Complications

No statistically significant differences were observed in the rates of readmission (7.4% vs. 7.2%, *p* = 0.912) and reoperation (2.5% vs. 2.0%, *p* = 0.559) between groups in the matched cohort. However, the older cohort demonstrated a significantly higher rate of non‐home discharge (71.7% vs. 87.6%, *p* < 0.001) and mortality (1.8% vs. 4.6%, *p* = 0.006) after matching (Table 4).

The overall rate of medical complications did not differ significantly between the age groups (9.4% vs. 12.4%, *p* = 0.097), although the older cohort trended toward a higher rate of medical complications after matching. However, myocardial infarction was significantly more prevalent in the older cohort (0.8% vs. 2.3%, *p* = 0.037) after matching. Overall surgical complications did exhibit a significant difference between age groups, with the older group having higher rates of complications (32.3% vs. 40.9%, *p* = 0.002). This difference was largely driven by blood loss requiring transfusion, which was notably higher in the 80+‐year‐old group (31.6% vs. 39.9%, *p* = 0.003) (Table 4).

**TABLE 4:** 30‐day outcome measures and complications after matching.

Outcome variable	60–79 years old (*n* = 607)	80+ years old (*n* = 607)	*χ* ^2^	*p*
Readmission	45 (7.4%)	44 (7.2%)	0.012	0.912
Reoperation	15 (2.5%)	12 (2.0%)	0.341	0.559
Non‐home discharge	435 (71.7%)	532 (87.6%)	47.823	**<0.001**
Mortality	11 (1.8%)	28 (4.6%)	7.656	**0.006**
Surgical complications				
Overall	196 (32.3%)	248 (40.9%)	9.602	**0.002**
Superficial surgical site infection	5 (0.8%)	7 (1.2%)	0.337	0.773
Wound infection	606 (99.8%)	604 (99.5%)	1.003	0.624
Deep surgical site infection	1 (0.2%)	0 (0.0%)	1.001	>0.999
Dehiscence	0 (0.0%)	1 (0.2%)	1.001	>0.999
Blood loss requiring transfusion	192 (31.6%)	242 (39.9%)	8.965	**0.003**
Medical complications				
Overall	57 (9.4%)	75 (12.4%)	2.754	0.097
Pneumonia	18 (3.0%)	22 (3.6%)	0.414	0.520
Unplanned intubation	8 (1.3%)	7 (1.2%)	0.068	0.795
Pulmonary embolism	5 (0.8%)	10 (1.6%)	1.688	0.299
Ventilator >48 h	5 (0.8%)	1 (0.2%)	2.680	0.218
Renal insufficiency	1 (0.2%)	1 (0.2%)	0.000	>0.999
Renal failure	1 (0.2%)	1 (0.2%)	0.000	>0.999
Urinary tract infection	24 (4.0%)	28 (4.6%)	0.321	0.571
Cerebrovascular accident	3 (0.5%)	3 (0.5%)	0.000	>0.999
Cardiac arrest	2 (0.3%)	5 (0.8%)	1.293	0.452
Myocardial infarction	5 (0.8%)	14 (2.3%)	4.331	**0.037**
Deep venous thromboembolism	5 (0.8%)	5 (0.8%)	0.000	>0.999
Sepsis	5 (0.8%)	7 (1.2%)	0.337	0.562
Septic Shock	2 (0.3%)	4 (0.7%)	0.670	0.687
C. diff infection	2 (0.3%)	3 (0.5%)	0.783	0.676

*Note*: *p*‐values less than 0.001 were outputted as <0.001 on the SPSS statistical package (in bold).

### Associated Risk Factors

In terms of readmission, factors that conferred significantly elevated relative risks of complications in the 30 days after surgery included a BMI less than 18 (relative risk [RR] 3.092, 95% confidence interval [CI]: 1.138–8.406), prior use of dialysis (RR 5.794, 95% CI: 1.533–21.888), and patients classified as ASA 3 (RR 2.819, 95% CI: 1.260–6.306) or ASA 4 (RR 5.593, 95% CI: 2.195–14.251).

Obesity class III and those whose initial operative times exceeded 180 min were associated with reoperation, with a relative risk increase of 3.649 (95% CI: 1.219–10.922) and 3.712 (95% CI: 1.445–9.536), respectively (Table 5).

**Table 5:** Risk factors associated with 30‐day outcome measures/complications

Readmission	Relative risk [95% CI]
Underweight BMI	3.092 [1.138–8.406]
Dialysis	5.794 [1.533–21.888]
ASA 3	2.819 [1.260–6.306]
ASA 4	5.593 [2.195–14.251]
Reoperation	
Obesity class III	3.649 [1.219–10.922]
Operative time >180 min	3.712 [1.445–9.536]
Non‐home discharge	
Obesity class II	1.731 [1.030–2.910]
Bleeding disorder	1.790 [1.017–3.150]
ASA 2	0.712 [0.508–0.998]
Mortality	
CHF	7.072 [1.355–36.909]
Wound infection	6.667 [2.393–18.576]
ASA 4	2.982 [1.325–6.709]
Surgical complications	
Wound infection	2.754 [1.410–5.379]
ASA 2	0.322 [0.202–0.512]
Operative time >120 min	1.947 [1.512–2.508]
Medical complications	
Wound infection	2.335 [1.041–5.241]
ASA 2	0.392 [0.212–0.723]

Obesity class II (RR 3.649, 95% CI: 1.030–2.910), bleeding disorders (RR 1.790, 95% CI: 1.017–3.150), and ASA class 2 (RR 0.712, 95% CI: 0.508–0.998) were found to be predictive of non‐home discharge.

CHF (RR 7.072, 95% CI: 1.355–36.909), wound infection (RR 6.667, 95% CI: 2.393–18.576), and ASA 4 (2.982, 95% CI: 1.325–6.709) were associated with overall 30‐day mortality.

General increases in surgical complications were found to be significantly increased in those with wound infection (RR 2.754, 95% CI: 1.410–5.379) and operative time exceeding 120 min (RR 1.947, 95% CI: 1.512–2.508) and notably decreased in those with ASA class 2 (RR 0.322, 95% CI: 0.202–0.512).

The relative risk of medical complications were found to be increased in patients with wound infection (RR 2.335, 95% CI: 1.041–5.241) and decreased in ASA class 2 (RR 0.392, 95% CI: 0.212–0.723).

## Discussion

### Summary of Main Findings

In summary, 30‐day outcomes and complications revealed significant disparities between the 60–79 and 80+ age groups, with the latter demonstrating higher rates of readmission, non‐home discharge, blood loss requiring transfusion, and death. Propensity score matching facilitated a more controlled comparison between the age groups, confirming the persistent elevated risk for mortality and non‐home discharge in the older cohort. The older cohort also experienced a higher rate of myocardial infarction, but it is unclear if this elevated risk is related directly to age or, rather, a higher level of comorbidity that is not captured within the variables available in the database. Within the entire population, several risk factors were independently associated with adverse outcomes. Notably, ASA classification, BMI status, overall operative time, dialysis use, and CHF emerged as determinants of poor 30‐day outcomes and complications. These findings underscore the vulnerability of elderly patients to adverse postoperative events, emphasizing the need for tailored management strategies. Understanding independent risk factors for various 30‐day complications might provide a starting point for discussions of risk stratification and shared decision‐making between providers and patients prior to surgical intervention for geriatric distal femur fractures.

### Distal Femur Fracture Population Characteristics

Distal femur fractures constitute approximately 0.5% of all fractures and 3% of femur fractures, with a notable increase in incidence after the age of 60.^9^ Although relatively less common injuries, they pose a substantial challenge to healthcare providers given their association with increased morbidity and mortality.^10^ Despite this, there is a relative lack of large population studies investigating short‐term outcomes and morbidity following distal femur ORIF, with existing research limited by small sample size and comorbidities. This study aimed to address these issues by harnessing the statistical power of a validated national database. In doing so, we clarified the incidence and risk factors for short‐term morbidity, mortality, and complications after distal femur ORIF in the geriatric population.

Notable characteristics in the population were observed in terms of gender and comorbidities. Most patients were women and often had a significant medical comorbidity burden. Female predominance in our cohort aligns with incidence of distal femur fracture in the literature, in part due to gender‐specific risk factors that increased the risk of fragility fractures.^11^ Our cohort also expectedly had a significant burden of medical comorbidities. In an epidemiologic study of all femur fractures, Nieves *et al*. demonstrated that patients with a fragility fracture of the distal femur had significant levels of medical comorbidity: roughly 21% had concurrent diabetes, 32%–44% had cardiovascular disease, and 5%–8% had renal failure or used dialysis.^12^ These percentages were comparable and often greater than those found in patients with fragility hip fractures in that same cohort.^12^ The demographics of our study suggested that the prevalence of these comorbidities might be even higher, with roughly 27% of our patients having diabetes and 75% having either CHF or hypertension. These associations underscore the importance of a comprehensive preoperative assessment to address and optimize modifiable risk factors.

### Mortality after Distal Femur Fractures

Mortality following geriatric distal femur fractures, regardless of management strategy, is substantial.^4^, ^10^, ^13^, ^14^, ^15^ Prior literature has demonstrated that geriatric distal femur fractures have at least comparable,^4^, ^13^ if not greater,^14^ mortality relative to hip fractures. Our study adds to the current literature by utilizing propensity score matching and multivariate regression to specifically investigate age as a risk factor for perioperative complications using a large sample size cohort from a validated surgical database. We specifically investigated the risk of complications in patients aged 80 years or older underoing distal femur ORIF. In our study, the total 30‐day mortality rate was 3.6%, which is slightly lower than those in the aforementioned studies. However, the overall 30‐day mortality rate in the 80+ year‐old group was 6.2%, nearly triple that of the 60–79‐year‐old group. Furthermore, the mortality rate was found to be significantly higher in the 80+‐year‐old group after controlling for medical comorbidities, BMI, and ASA classification with propensity score matching. Being aware of the difference in mortality within the geriatric population prompts an evaluation of risks of surgical intervention in octogenarians and will allow healthcare providers to communicate realistic expectations and potential risks to their patients.

### Reoperation after Distal Femur Fractures

The risk of reoperation is a significant consideration for geriatric distal femur fractures. Nonunion, infection, and hardware failure can be catastrophic complications for any patient, but in particular elderly patients with medical comorbidities. Jennison *et al*. reported that 9.1% of patients with a distal femur fragility fracture required at least one subsequent operation after the index procedure.^13^ The most common indications for further operations were periprosthetic fractures, hardware failure, and infection. A multicenter study by Ricci *et al*. found that 2% of geriatric distal femur fracture patients srequired reoperation to promote union, with greater risk of reoperation associated with smoking, diabetes, and younger age.^16^ In a cohort of 176 patients with low‐energy distal femur fractures managed with lateral‐based locked plating, Moloney *et al*. reported a nonunion rate of 24%, with most patients requiring revision surgery.^17^ Reoperation rates in our study were overall consistent with this prior literature, but we also identified obesity and prolonged operative time to be predictive of reoperation.

Importantly, we only investigated reoperation rates after distal femur ORIF. However, there are certainly alternative surgical techniques for managing these injuries, often dictated by the fracture pattern but also, to some extent, the overall medical comorbidity of the patient.^18^ For example, Hart *et al*. demonstrated that functional outcomes might be improved after distal femur replacement relative to ORIF for comminuted distal femur fractures in the elderly population, with similar rates of 1‐year mortality.^6^ There is need for further investigation in this domain, particularly with high‐quality prospective studies and randomized controlled trials.

### Limitations and Prospects

While providing substantial statistical power, use of the NSQIP database introduces certain limitations that warrant consideration. The inherently retrospective design might lead to potential selection bias. In terms of cohort makeup, the database's bias toward data from large academic hospital systems might also affect the generalizability of our findings to other healthcare settings. In terms of data completeness, while the 30‐day follow‐up captures immediate postoperative outcomes and complications, it limits our understanding of longer‐term consequences and the potential for delayed complication. Additionally, the NSQIP database lacks injury‐ and surgery‐specific parameters, such as AO fracture classification, bone quality considerations, and soft tissue compromise. These are important for comprehensive analysis of distal femur fractures, which have a wide range of severity. There is also an absence of detailed information on the severity of medical comorbidities, individual complications, and radiographic outcome measures, which further limits the granularity of the conclusions. Despite these limitations, this study clarifies 30‐day morbidity and mortality after distal femur ORIF in the geriatric population, particularly in octogenarians and older patients. Future investigation might consider prospective study design or leverage large insurance databases with extended follow‐up periods to provide a more robust understanding of distal femur fracture outcomes over time, thus allowing us to better address poor outcomes.

## Conclusion

Geriatric patients undergoing ORIF for distal femur fractures are at significant risk for 30‐day morbidity and mortality. After controlling for medical comorbidities, octogenarian and older patients specifically are at increased risk for mortality, non‐home discharge, and surgical complications relative to patients aged 60–79 years old. Multiple variables including BMI, ASA classification, operative time, and certain medical comorbidities, are independently associated with poor short‐term outcomes. These findings might be useful for risk stratification and shared decision‐making discussions with patients and their families prior to surgical intervention.

## Author Contributions

JV wrote the paper and performed the analysis. MAP wrote portions of the paper, conceived and designed the analysis, collected the data, and performed the analysis. EBG assisted with performing the analysis and assisted with writing tables/the manuscript and critical edits. TC assisted with writing tables/the manuscript and critical edits. JD assisted with writing tables/the manuscript and critical edits. CW assisted with writing tables/the manuscript and critical edits. BB was involve in the conceptualization of study, management, critical edits of the manuscript, and project oversight.

## Conflict of Interest Statement

The authors have no relevant financial disclosures related to the content included in this manuscript.

## Funding Information

No external or internal funding was used for the purposes of this study.

## Data Availability

The American College of Surgeons National Surgical Quality Improvement Program and the hospitals participating in the ACS NSQIP are the source of the data used herein; they have not verified and are not responsible for the statistical validity of the data analysis or the conclusions derived by the authors.
